# Correspondence

**Published:** 1872-11

**Authors:** Dewitt C. Crumb

**Affiliations:** Preston, N. Y.


### Correspondence.

PROF. MINER, *Editor Buffalo Medical and Surgical Journal.*

SIR,—I have the pleasure of communicating the following (to me) very peculiary case: Mr. J. G——, a native of Ireland, called my attention to his case about two weeks ago. He gave me his past history as follows: Has been a laborer on public works all his time since childhood, has drank to excess at times, has been in this country fifteen years, had scrofula several times, in fact, there were a dozen cicatrices to be seen in the sub-maxillary and inguinal region alone. He has had a cough about a year and expectoration seven or eight months of the time. On the 13th of June last a small abscess formed upon the chest between the first and second ribs on the right side, and close to the sternum. It discharged pus, and still continued to do so until his death, the quantity gradually increasing. When I first saw him the right shoulder dropped nearly three inches, the sternal end of the clavicle was disarticulated and drawn up about one and half inches.

In the first intercostal space, about one inch to the right side of the sternum, was an opening one-half inch in diameter, with a discharge of pus and air at every expiration. There was dullness over both lungs. Auscultation denoted softened tubercles and cavities.

Patient died Oct. 21, 1872. Sectio cadaveris, twenty hours after death. Sawed through the ribs upon both sides and lifted the whole frame of the thoracic walls. Upon the upper and anterior part of the right lung there was pleuritic adhesions enclosing a large vomica, into which the sinus opened. Freeing this from pus I found the right bronchial tube, about three inches from its entering the lung, passing entirely through this opening or vomica. Both lungs were thickly studded with softened tubercles. There was several vomica to be seen in the left. The clavicle was disarticulated at its sternal end, and drawn up by the contraction of the sterno-cleido-mastoid muscle. About one of the first rib was gone, and the right border of the sternum at the articulations of the clavicle and first rib was carious. Tuberculosis we are all too familiar with, but to have the most of the discharge from the lung through a sinus leading to a bronchial tube is a rare anomally so far as my knowledge extends upon such matters.

Comments on my part are unnecessary.

Respectfully yours, DEWITT C. CRUMB.

Preston, N. Y., Oct. 24, 1872.

